# Non-NMDA Mechanisms of Analgesia in Ketamine Analogs

**DOI:** 10.3389/fpain.2022.827372

**Published:** 2022-02-15

**Authors:** Logan J. Voss, Martyn G. Harvey, James W. Sleigh

**Affiliations:** ^1^Anaesthesia Department, Waikato District Health Board, Hamilton, New Zealand; ^2^Emergency Department, Waikato District Health Board, Hamilton, New Zealand

**Keywords:** ketamine, analgesia, NMDA – N-methyl-D-aspartate, potassium channels, psychomimetic

## Abstract

Despite 50 years of clinical use and experimental endeavor the anesthetic, analgesic, and psychomimetic effects of ketamine remain to be fully elucidated. While NMDA receptor antagonism has been long held as ketamine's fundamental molecular action, interrogation of bespoke ketamine analogs with known absent NMDA binding, yet profound anesthetic and analgesia fingerprints, suggests alternative targets are responsible for these effects. Herein we describe experimental findings utilizing such analogs as probes to explore ketamine-based analgesic molecular targets. We have focused on two-pore potassium leak channels, identifying TWIK channels as a rational target to pursue further. While the totality of ketamine's mechanistic action is yet to be fully determined, these investigations raise the intriguing prospect of separating out analgesia and anesthetic effects from ketamine's undesirable psychomimesis—and development of more specific analgesic medications.

## Introduction

Ketamine is a dissociative anesthetic and potent analgesic. Its well-documented molecular action as a non-competitive NMDA channel antagonist has long been viewed as the likely mediator of its anesthetic and analgesic properties (1). However, this assumption has come under increasing scrutiny with the realization that ketamine affects multiple molecular targets within the central nervous system (2, 3). Even so, and despite more than 50 years of clinical use, our understanding of ketamine's molecular level mechanisms of action remains inadequate. This severely stymies “soft” designer drug development targeting ketamine's analgesic and anesthetic profile whilst minimizing its undesirable hallucinogenic properties. The latter is particularly relevant for ketamine's prescription as an analgesic, because effective dosing for analgesia overlaps with its psychotomimetic dose response profile (4).

**Figure 1:**
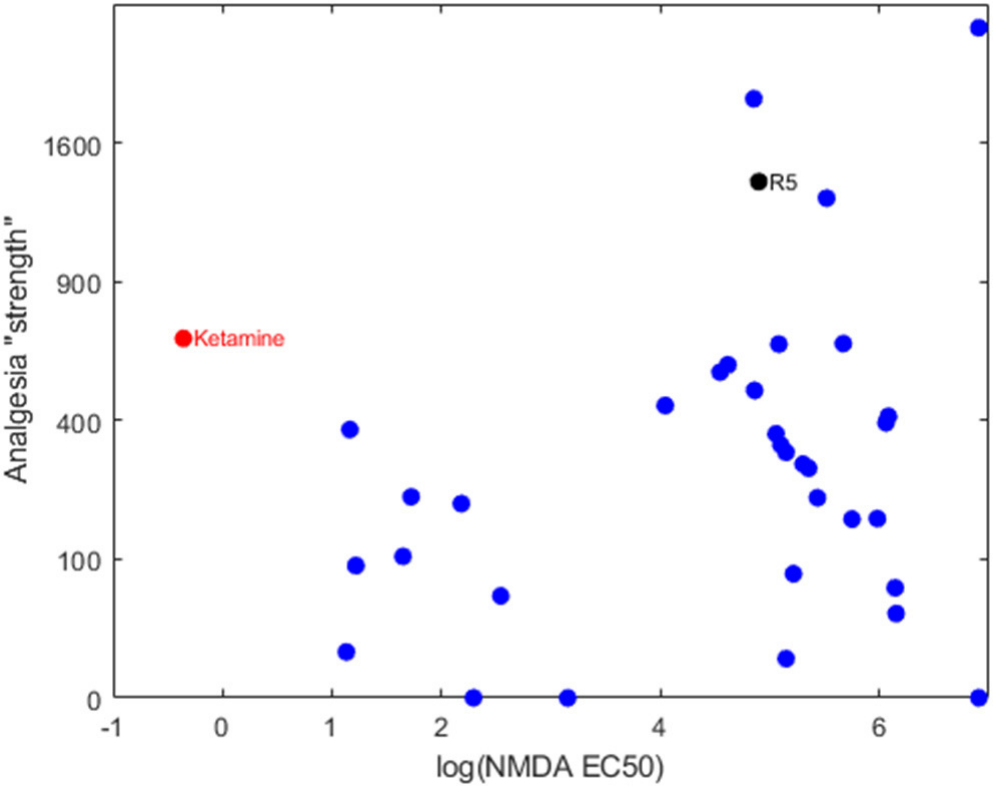
Bi-logarithmic plot of NMDA binding (EC50) vs. analgesia for a series of ketamine analogs. The red dot is the parent ketamine, the black dot is R5 and the blue dots are other ketamine-ester variants. There is no correlation, suggesting that NMDA has a negligible role in production of analgesia for these compounds. Determination of NMDA inhibition was carried out by commercial provider (Eurofins Panlabs Taiwan, Ltd. Pharmacology Laboratories) according to reported procedure (7). MK-801 {[5*R*,10*S*]-[+]-5-methyl-10,11-dihydro-5*H*-dibenzo [*a*,*d*]cyclohepten-5,10-imine}, Ketamine and ketamine analogs were tested for competitive binding against the radioligand 5 nM [^3^H]-MK-801 in wistar rat brain preparations, incubated in 5 mM Tris-HCL at pH 7.4 for 3 h at 25°C. Radioligand displacement was used to determine ligand binding affinity to calculate IC_50_.

Our group has been developing and testing bespoke ester analogs of ketamine—with the aim of circumventing ketamine's hallucinogenic side effects by facilitating rapid hydrolysis to inactive carboxylic acid metabolites (5). The hypothesis is that the psychotomimetic window would be shortened due to the rapid degradation of the active compound by tissue esterases. Serendipitously, the family of 30+ analogs were found to possess a broad range of NMDA receptor binding affinities. When the NMDA affinities are related to their analgesic potencies, it's clear that the analgesic component is not explained by an NMDA blocking action (6) (see Figure 1). One compound in particular, an isopropyl ester (“R5,” “SN35563”) with similar analgesic potency to ketamine (5) has an NDMA affinity 191 times less than ketamine (8). This confirms beyond any doubt that ketamine-based analgesia can occur independent of its NMDA antagonism. This raises the tantalizing prospect of investigating analgesia mechanisms in a ketamine model void of NMDA activity—and development of more specific analgesic medications if drug targets are known.

These discoveries opened the door to using R5 as an experimental tool for investigating possible molecular targets for ketamine analgesia, without the confounding influence of NMDA antagonism. With an almost endless number of potential targets, we have focused primarily on two-pore potassium “leak” (K2P) channels. Potassium channels are ubiquitous in all living organisms and in humans comprise at least 80 different subtypes. K2P channels, in particular, form a 15-member subclass that provide background neuronal currents responsible for stabilizing the resting membrane potential (9). Our attention was drawn to K2P channels for two reasons. Firstly, K2P channels are emerging as important potential targets for nociceptive regulation (10). Secondly, and more importantly, K2P channels have been previously implicated in ketamine's mechanism of action (11). In the sections that follow we will outline a series of *ex vivo* and *in vitro* studies exploring K2P channels as a possible ketamine analog (R5) target.

## *Ex vivo* Brain Slice Effects of R5

### Cerebral Cortex

We first established that R5 behaved similarly to ketamine in *ex vivo* mouse cortical slices. Ketamine strongly inhibits cortical seizure-like event (SLE) activity, an effect that is barium chloride (BaCl_2_) and urethane sensitive—implying involvement of K2P channels (11).

The methodology for slice preparation, electrophysiological recording and pharmacology manipulation is detailed elsewhere (11). Briefly, 400 μm coronal mouse brain slices were prepared from adult male and female C57 mice. The tissue slices were prepared in HEPES-buffered “normal” artificial cerebrospinal fluid (aCSF) (oxygenated with 95% oxygen), before being transferred to a submersion-style perfusion bath replenished with oxygenated aCSF void of magnesium ions (“no-Mg aCSF”) by gravity-feed at a rate of 5 mL/min. The lack of magnesium ions in solution activates the tissue by unblocking NMDA receptors, resulting in the generation of repeating population bursts known as seizure-like events (SLEs). Spontaneous extracellular field potentials were recorded from layer IV of the somatosensory cortex, amplified and filtered (low pass 300 Hz and high pass 1 Hz) and stored for later analysis after analog-digital conversion. All test drugs were added directly to pre-oxygenated no-Mg aCSF to the required concentrations. The main outcome variable was the change in SLE inter-event frequency, which is reliably reduced by ketamine and other anesthetics (11, 12). Because of degradation of R5 by tissue esterases, a much higher R5 concentration (100 μg/mL) than ketamine (4 μg/mL) was required to achieve a similar reduction in SLE frequency. The pan carboxyl-esterase inhibitor 2,2′-Thenil (13) confirmed that esterase activity was largely responsible for this relative lack of potency, restoring R5 effectiveness in this model at 15 μg/mL, while having no effect on ketamine action at 4 μg/mL.

Potassium channel effects of R5 were probed using BaCl_2_ and urethane. Urethane is a central nervous system depressant (anesthetic) that activates barium-sensitive potassium leak channels (14). BaCl_2_ at low concentrations (<200 μM) blocks potassium leak currents, with TREK and TWIK-mediated currents particularly implicated (14–16). Effects were similar to those reported for ketamine, where SLE frequency reduction was blocked by barium chloride and enhanced by urethane (11). BaCl_2_ (200 μM) eliminated the R5 suppression of SLE frequency. At a concentration sufficient to significantly reduce cortical neuronal spike rates *in vitro* [>10 mM (14)], we found that urethane (20 mM) reduced SLE frequency by 27 (13)%, but the reduction was significantly increased to 60 (17)% when combined with R5. In keeping with a potassium leak channel enhancing effect, reducing the potassium concentration in the aCSF enhanced the effect of R5. The data implicate opening of barium-sensitive TREK and/or TWIK K2P channels in the cortical depressant effect of R5 in cortical slices. This was examined further with the TREK-1 blocker, spadin (18). At two concentrations (7 and 25 μM), spadin had no effect in isolation, nor did it modify the effect of R5 on SLE frequency—seemingly eliminating cerebrocortical TREK-1 channels as a target.

### Differential Sensitivity, Basolateral Amygdala vs. Cerebral Cortex

Subanaesthetic concentrations of ketamine have been reported to have both cortical and subcortical effects (19). In an earlier transcriptome study we identified two central subcortical nuclei that were particularly sensitive to R5 for large-scale gene expression changes, the basolateral amygdala (BLA) and paraventricular nucleus of the thalamus (PVT) (20). This suggested that R5 was likely to be acting centrally with differential sensitivity at specific subcortical nuclei. To test this in mouse brain slices, cortical slices were prepared as above and selected for those containing the BLA. No-Mg SLE activity was recorded concurrently from the auditory cortex and the BLA by overlaying a 2 × 16 rectangular electrode array (50 μm stainless steel, 500 μm row separation, and 250 μm electrode separation). Extracellular field potentials were recorded using a Blackrock Cereplex system (high pass filter 0.3 Hz and low pass filter 250 Hz) with 1 kHz sampling.

After baseline recording to establish stable SLE activity, R5 was perfused (5 ml /min) for 20 mins at a time at sequential increasing concentrations of 20 and 30 μg/mL and for 10 min at 40 μg/mL. Because SLE parameters (frequency, length, and amplitude) appeared to be affected differentially, a composite measure equating to SLE frequency ^*^ length ^*^ amplitude was quantified for each recording to give a measure of overall activity (normalized to percent change from baseline).

**Figure 2:**
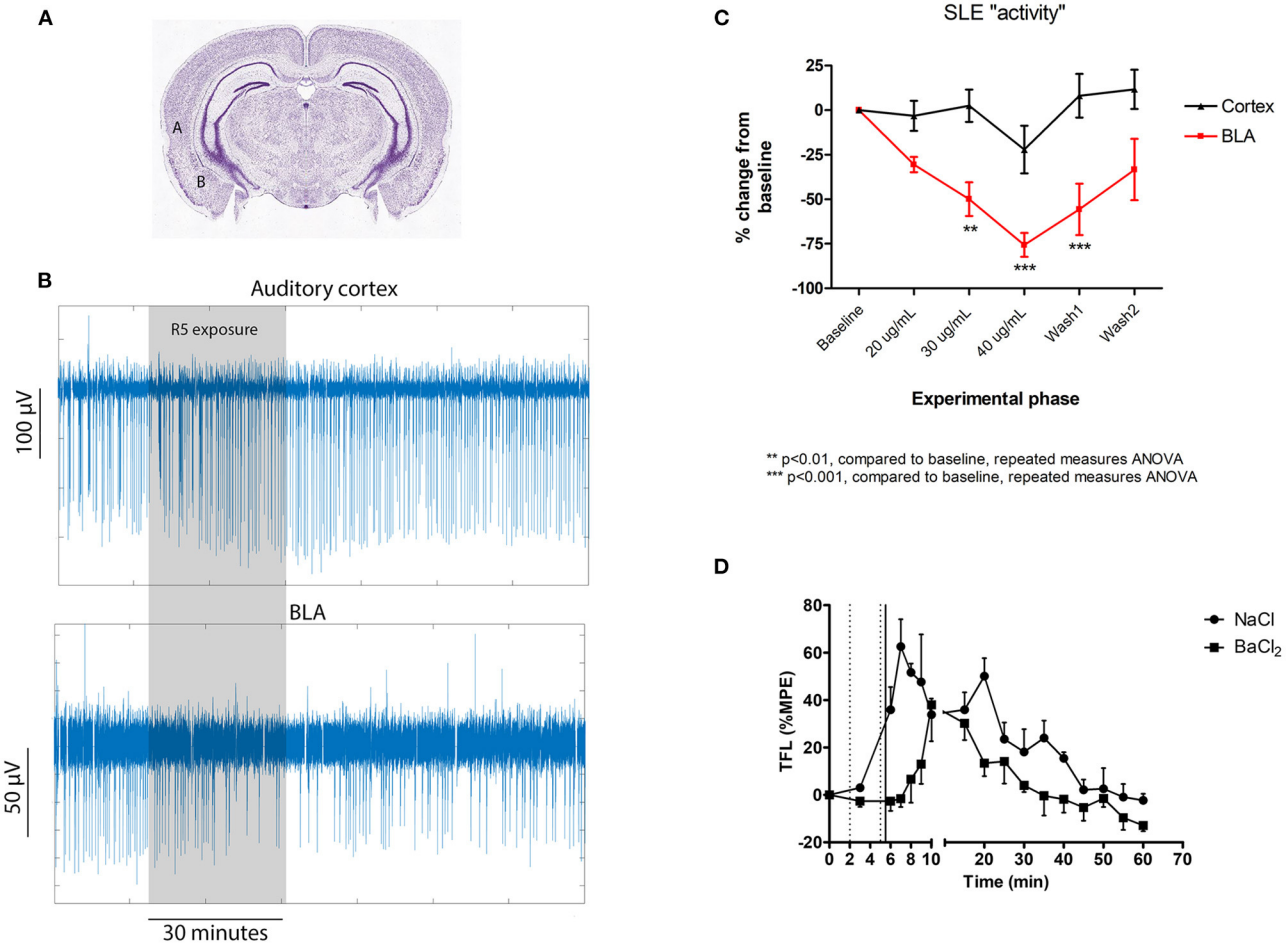
Central subcortical effects of R5 in mouse cortical slices **(A–C)** and following intraventricular microinjection in rats **(D)**. **(A)** Cortical slice recording locations for comparing the effect of R5 on auditory cortex (labeled A) and basolateral amygdala (BLA- labeled “B”); **(B)** representative cortical slice recording traces and **(C)** slice group data comparing auditory cortex and BLA; **(D)** tail flick latency (TFL) as %MPE recordings according to intraventricular injection group. Dotted vertical lines represent saline/ BaCl_2_ injection. Solid vertical line represents R5 injection. 2-way RMANOVA *p* = 0.005; max difference at 6, 7, 8, 9 min *p* < 0.001 all.

SLE activity was synchronous across the cerebral cortex and the BLA, consistent with findings showing that the auditory cortex interacts directly with the BLA in fear learning (21). The clear finding was that BLA activity was significantly more sensitive to R5 than cortical activity (Figure 2). In fact, no significant change was identified in the cortex, while a clear reduction was noted in the BLA from the 30 μg/mL time point. The BLA effect reversed by the end of a 40 min wash period.

Together, the brain slice results suggest that R5 acts centrally and with differential sensitivity at cortical and subcortical locations. The BLA, which is involved in fear conditioning to aversive stimuli (22) may be specifically implicated. *In vivo* “adhesive removal test” (23) experiments strengthen the case for involvement of the limbic system in R5 analgesia, showing that rats administered subanaesthetic doses of R5 were behaviourally normal, but oblivious to what would ordinarily be the noxious stimulus of having a small piece of sticky tape adhered to a forepaw.

The BLA was chosen over the PVT for electrophysiological study because of its suitability for investigation using our established low-magnesium cortical slice model. That is, the BLA is interconnected with the auditory cerebral cortex (21) and generates low-magnesium seizure-like event activity that is synchronized (connected) with activity recorded from the auditory cortex. This allowed for concurrent recording from both regions and direct pairwise comparison of the R5 effect. We would emphasize that this does not preclude the PVT, or potentially other subcortical nuclei, from important mechanistic roles in the action of R5. It should also be noted that the cortical slice model has been principally validated against the hypnotic effects of anesthetic drugs—including the family of ketamine analogs (24). The correlation between ketamine analogs and *in vivo* hypnotic potency and SLE slice effect is robust (*R*^2^ = 36%, *p* = 0.003), but is much weaker for the analgesic effect (*R*^2^ = 17%, *p* = 0.06). The bearing of these findings on mechanisms of ketamine analgesia must therefore be substantiated in a more relevant model. The section that follows describes *in vivo* experiments designed to confirm a central K2P mechanism in a validated analgesia model.

## Intraventricular Microinjection of R5 and Effect of Barium Chloride

Drug microinjection is a technique for the direct mechanical delivery of small drug volumes to specific stereotaxically identified brain regions (25). Because the BLA (and PVT) appear to be highly sensitive to R5, we took advantage of both nuclei having paraventricular aspects (the lateral ventricles and third ventricle for BLA and PVT, respectively) and quantified the effect of intraventricular R5 microinjection on pain responses in rats. Tests were repeated with barium chloride pretreatment. Based on the R5 brain slice findings, we hypothesized that BaCl_2_ would antagonize a central analgesic effect of R5. Rats were used for this investigation because previous behavioral investigations had utilized the rat model (5, 6, 8, 26). The reason for this is the necessity for secure intravenous tail vein access for drug delivery, which limits drug metabolism by tissue esterases (27). Tail vein drug delivery is not practicable in the smaller mouse model.

Chronically instrumented adult female Sprague Dawley rats were studied. For direct brain cannula implantation rats were anesthetized with isoflurane 2% then stereotaxically implanted unilaterally (right) with one 26-gauge cannula into the anterior horn of the lateral ventricle. Cannulas were held in place with dental cement, and patency maintained with occlusive stylets. A tail flick analgesia meter (Colombus Instruments, Colombus, Ohio) was used to determine pain sensitivity. Radiant heat was applied using a shutter-controlled lamp as a heat source focused on a spot located 6–8 cm from the tip of the tail. The intensity of the beam was set at a level producing basal latency times between 3 and 4.5 s. To prevent thermal tissue injury the cut off time was set at 10 s. A digital response time indicator with a resolution of 0.1 s measured the time from initiation of stimulus until tail withdrawal (the flick). Tail flick latency (TFL) was calculated as a percentage of the maximum possible effect (MPE) such that:


$$\begin{array}{l} \%\text{MPE = }[\text{TFL }(\text{post - drug})\text{ - TFL}(\text{pre - drug})\text{/}\\ \text{ 10s - TFL}(\text{pre - drug})]\times \text{100}\% \end{array}$$

For intraventricular injections, solutions were loaded into 30 cm lengths of PE-50 tubing attached at one end to a 10 μL Hamilton syringe prefilled with test agents. All injections were delivered by 1.0 μL bolus over 30 s. Following assessment of baseline tail flick latency (time 0) rats underwent intraventricular injection of 1 μL saline (0.9%) or 1 μL BaCl_2_ (10 mg/mL) at time equals 2 min. TFL was reassessed at 3 min. Further 1 μL injections of saline or barium solutions were undertaken at 5 min and followed immediately by intraventricular injection of 1 μL R5 (10 mg/mL). TFL evaluation was undertaken at 1 min intervals from time 6 to 10 min, then at 5 min intervals thereafter to 60 min.

TFL responses as %MPE are presented in Figure 2D. Intraventricular microinjection of R5 had a clear analgesic effect, seen as a significant increase in tail flick latency lasting for nearly 60 min. Importantly, pretreatment with BaCl_2_ antagonized the early phase of R5 analgesia, delaying the increase in TFL by 8–10 min. The data confirms the cortical slice results showing that R5 analgesia is centrally mediated and barium-sensitive. While both the BLA and PVT have paraventricular regions, many other nuclei could have been affected by intraventricular R5 injection. These results do not exclude involvement of other regions such as the periaqueductal gray and pretectal areas. A more targeted suite of experiments will be necessary to confirm involvement of specific nuclei.

## Electrophysiological Quantification of TREK-1 and TREK-2 Effects of R5

The cortical slice work suggested that TREK-1 K2P receptors were not relevant to the cortical depressant effect of R5. To unequivocally rule out TREK channels as an R5 target, recombinant TREK-1 (K_2P_2.1) and TREK-2 (K_2P_10.1) expressing cells (host cell mouse HEK) were electrophysiologically tested using the voltage clamp technique in patch clamped whole cells.

Outward potassium currents were measured at voltage steps from −80 to +100 mV (300 ms). The testing protocol consisted of first adding extracellular physiological solution (control period) twice (2–3 min), followed by one concentration of test compound (2–3 min) and a saturating inhibitory concentration of specific channel blocker BaCl_2_ (10 mM). For all test compounds, nine concentrations were tested, 0.1, 0.3, 1, 3, 10, 30, 100, 300, 1,000 μM. Data were normalized using the last point obtained during the pre-compound saline application as the zero value (0) and maximum block by the reference blocker (BaCl_2_) as the top value (1.0).

All cells used in these experiments showed functional TREK-1 and TREK-2 channels which were blocked by the addition of a high concentration of Tetrahexylammonium (THA) and Quinidine, respectively, validating the assay. R5 induced a weak concentration dependent block of TREK-1 and TREK-2 channels, with an IC_50_ of 24 μg/mL for TREK-1 and 104 μg/mL for TREK-2. Ketamine was also tested and had no effect on TREK-2 and a very weakly blocked TREK-1 (EC_50_ 118 μg/mL). There are two telling observations to make from these results. Firstly, the EC_50_ values for the TREK channel effects were up to 2 orders of magnitude more than the therapeutic concentrations for these agents. Secondly, the effect noted was channel blockade—whereas channel opening was hypothesized to explain the R5 depressant effect. Based on these results, TREK K2P channels can be ruled out as a therapeutic target for R5 and ketamine.

## Other Potential Targets

As we've discussed, barium sensitivity at low concentrations (<200 μM) implies involvement of TREK or TWIK-mediated currents (14–16). With TREK channels effectively ruled out, TWIK channels remain the most likely K2P target for R5. Cesium chloride (CsCl) is ineffective at TWIK-1 channels in the low mM range (28), while barium (Ba^+2^) strongly blocks TWIK-1 currents in the μM range (28). Thus, Cs^+^ and Ba^+2^ can be used to test for TWIK-1 specificity. In our cortical slice experiments, 2 mM CsCl was ineffectual on R5, strengthening the case for TWIK channel involvement. Experimental electrophysiological verification of TWIK channel involvement in R5 and ketamine action remains to be undertaken.

While our focus in the presented studies has been K2P channels, numerous other potential analgesia targets could be relevant. We touched on a subset of these using commercial channel screens but failed to identify any R5 molecular targets at clinically relevant concentrations. These include radioligand binding studies on calcium L-type channels, GABA_A_ receptors and δ_1_ and μ opiate receptors—and patch clamp functional assay of HCN1 potassium channels. Binding profiles were sourced from Eurofins Panlabs Ltd. (Taipei, Taiwan). Duplicate studies were undertaken at concentrations 10 μM, 1 μM, 0.1 μM, and 10 nM, for the compounds. Detailed explanations of the methodology employed in potential-target profiling can be found at http://www.eurofinspanlabs.com/Panlabs using the number listed in parentheses below: calcium channel L-type, opiate δ1 (260130); opiate μ (260410); norepinephrine uptake (302000); and hyperpolarization-activated cyclic nucleotide-gated ion channel, HCN1 (952727). Greater than 50% binding inhibition was deemed significant. Reference standards were run as an integral part of each assay to ensure the validity of the results obtained.

## Discussion

The role of the NMDA receptors in ketamine analgesia has been largely unquestioned. This view has been strengthened by the likes of compounds such as phencyclidine and dizolcipine, which potently bind to the NMDA receptor complex and are strong analgesics (29). Of the ketamine enantiomers, S(+)-ketamine has about 3-fold more analgesic potency than R(-)-ketamine, which has been attributed to its increased NMDA affinity (30). Our data indicates that targets other than the NMDA receptor are important mediators of analgesia for ketamine analogs. It's likely that NMDA binding is only one component of a ketamine-related analgesic cascade, and that ketamine analog compounds have varying degrees of additional actions that contribute to the overall analgesic activity.

Based on relative sensitivity to barium, caesium, and urethane, the TWIK-1 channel is a plausible component of a ketamine-analog analgesic mechanism. TWIK-1 was the first mammalian K2P channel identified (31), so-called because it is a weakly inward rectifying two-pore potassium channel. It is widely expressed in the brain where, like other K2P channels, it is thought to provide a leak potassium conductance that stabilizes the membrane potential (17). Further work demonstrating positive allosteric modulation of the TWIK-1 channel by ketamine is required to confirm our findings. These are challenging experiments however, because heterologous expression of TWIK-1 proteins in Xenopus oocytes or cultured mammalian cells results in low channel functionality (32–34). This impediment to studying the functional characteristics of TWIK-1 may be one of the reasons this channel has not been favored to date as an anesthetic target (35). Our studies support a centrally located analgesic mechanism, the primary brain regions most likely linked to the limbic system—principally the baso-lateral amygdala and cerebral cortex. The implication here is that the analgesic potential of ketamine analogs may reside in their capacity to moderate the offensiveness of pain signals entering the brain, more than inhibit their propagation. This is contrary to the view that ketamine acts at least in part as a local anesthetic *via* NMDA blockade (36).

In summary, challenging the widely view that ketamine analgesia is due to NMDA blockade is the first step toward separating out analgesia from its undesirable psychomimetic side effects. Identification of analgesic molecular targets will aid the development of more potent and specific analgesic medications. We have used ketamine analogs that are void of both NMDA action and psychomimetic effects as model compounds to begin differentiating these effects. In particular, centrally expressed K2P channels should be carefully scrutinized as potential targets for ketamine analgesia.

## Data Availability Statement

The raw data supporting the conclusions of this article will be made available by the authors, without undue reservation.

## Ethics Statement

The animal study was reviewed and approved by University of Waikato Animal Ethics Committee.

## Author Contributions

LV wrote the manuscript and conducted the cortical slice experiments. MH conducted the *in vivo* experiments and wrote the manuscript. JS wrote the manuscript. All authors contributed to the article and approved the submitted version.

## Conflict of Interest

The authors declare that the research was conducted in the absence of any commercial or financial relationships that could be construed as a potential conflict of interest.

## Publisher's Note

All claims expressed in this article are solely those of the authors and do not necessarily represent those of their affiliated organizations, or those of the publisher, the editors and the reviewers. Any product that may be evaluated in this article, or claim that may be made by its manufacturer, is not guaranteed or endorsed by the publisher.
